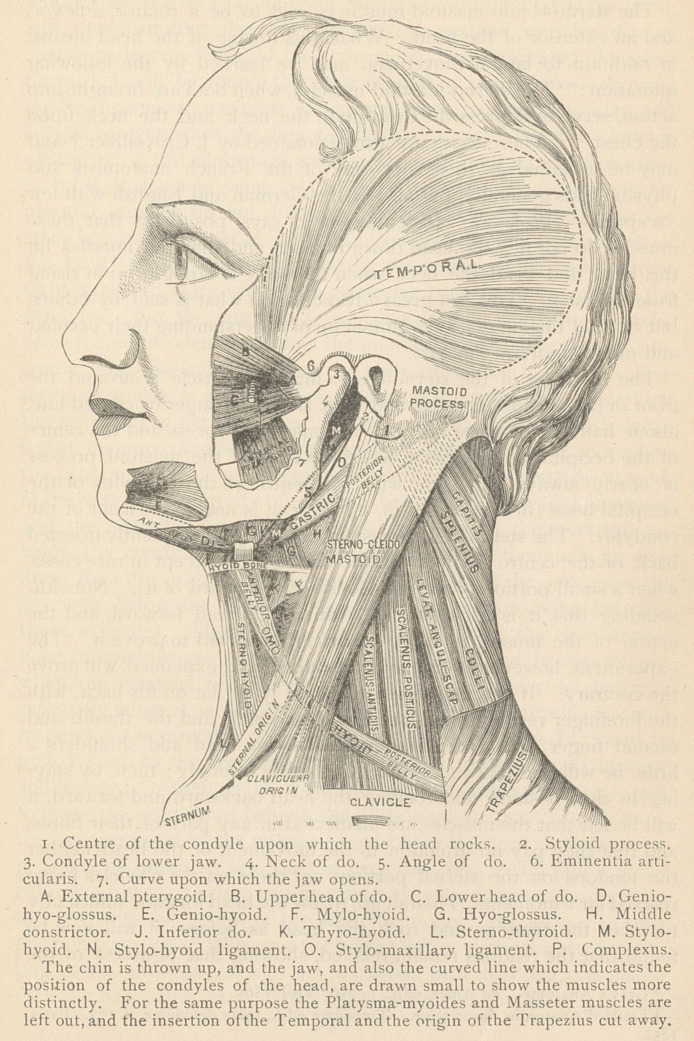# Treatment of Fracture of the Jaw, with Critical Remarks

**Published:** 1880-04

**Authors:** Thomas Brian Gunning

**Affiliations:** New York


Vol. I.——*April, 1880.*——No. 4.

ARTICLE I.

### TREATMENT OF FRACTURE OF THE JAW, WITH CRITICAL REMARKS.

BY THOMAS BRIAN GUNNING, D. D. S., NEW YORK.

I shall say very little of much that has been already well-told by others; but to prevent practitioners and students from being misled, I purpose to point out error when necessary.

Knowledge of what a bone has to do, and of the muscles which affect it, is indispensable when judging of its injuries; and if it is fractured, to determine the best means of holding the fragments in place. This paper will, therefore, show the use of the lower jaw, and correct the mistakes held in regard to the muscles which control it, and in other particulars. Diagnosis of its fractures will receive attention, and my own methods of controlling them be clearly shown.

The changes in form and condition of the lower jaw, from infancy to old age, are so well known and described that little need be said here, beyond calling attention to its obtuse angle in the earlier and the later periods of life compared with its adult form, which last gives greater freedom to its movements, while the neck of the condyle is more liable to be fractured, through being less in line with the chin, the most exposed part of the jaw. The condyle and its inter-articular cartilage sit close up in front of the ear when the mouth is closed, but they are brought forward under the rounded projection (*eminentia articularis*) to open the teeth.

The mobility of this joint is singularly unlike in different individuals. In some the condyle appears to move very much as in the carnivora, while in others it is so loosely attached that it is difficult to learn its proper position under unusual impressions. The jaw has, however, always more or less lateral movement in natural conditions, and its condyles always come forward to open the mouth. The variety and completeness of its action and movements fit it perfectly for the efficient use of its different forms of teeth and of those in the jaw above it. The human jaw has not, however, that extreme contrast of action to be found in that of some of the lower animals. In the beaver, neither this difference in its own movements, nor their approach to those of the human jaw, is, I think, in the least suspected. The rodents, according to Cuvier and Owen, have horizontal motion of jaw only from back to front and the reverse. Now this is true of the capibara, the largest rodent of South America, as its condyle moves in a groove which prevents lateral motion. But in the beaver, the largest living rodent of North America, the jaw in eating moves from side to side. In this rodent the temporal bone does not form any socket over the condyle, but it merely projects out in front of it, something like the *eminentia articularis* in the front of the glenoid cavity of the human temporal bone; while the auditory process of the beaver is very strong and curves forward so as to enclose the back of the condyle. By this formation, when the jaw is held forward, as in gnawing with the incisor teeth, the molars are well separated; whereas in eating with molar teeth the condyle is back against the bone of the ear, and the lower incisors being then clear of the upper, the muscles, by alternate action upon the sides of the jaw, work the condyles up and down on the temporal bone and thus swing the lower molars from side to side around the upper ones. Thus the beaver in eating moves its jaw at right angles to the movements in gnawing; the slight circularity of these motions being left unconsidered. It should be borne in mind that this rodent in eating, like other animals, including man, uses the teeth of one side only at the same time. In comparing the lateral movements of the beaver's jaw with those of the human maxilla, it will be seen that while the body of the beaver's jaw moves from side to side equally through its whole length, the lateral motion of the human jaw is greater at the canine and less in proportion as the part is nearer the condyle.

The use of the jaw can, however, only be well understood through acquaintance with the muscular action which affects it. This will now be fully explained.

The sterno-cleido-mastoid muscle is said to be a rotator, a flexor, and an extensor of the head. What this flexing of the head means, in addition to lateral movement, may be learned by the following quotation: "The sterno-mastoid muscles, when both are brought into action, serve to depress the head upon the neck, and the neck upon the chest."* These views are also maintained by J. Cruveilhier,† and may be accepted as those not only of the French anatomists and physiologists generally, but also of the German and English with few exceptions. Professor Henle, however, says positively that these muscles do not flex the head down in front, and that the muscles lift the head and bend the neck when the body is brought up in rising from the back. This is a great advance upon what is said by others, but beyond this he gives no intimation of understanding their peculiar and most important function.

The insertion of the sterno-cleido-mastoid muscle is around the front of the mastoid process, and back along the superior curved line, about half the distance between the mastoid process and the centre of the occipital protuberance, while the front of the mastoid process is nearly always on a line with the centre of the condyles of the occipital bone (in rare instances, however, it is nearer the front of the condyle). The sterno-cleido-mastoid muscle is consequently inserted back of the centre upon which the head rocks (except in rare cases, when a small portion of the muscle is a little forward of it). Notwithstanding this, it is set down as rocking the head forward, and the action of the muscle in rising is brought forward to prove it. The experiment, however, if properly conducted and explained, will prove the contrary. If the experimenter, while lying flat on his back, with the forefinger resting in the interclavicular notch, and the thumb and second finger on the tendons, will raise his head and shoulders a little, he will find that the muscles are acting strongly; then, by staying in that position and rocking the head backward and forward, it will be felt that the muscles are unaffected in any part of their fibres, and that they pay no attention to the movement of the head, neither the tendons on the sternal portions nor those on the clavicles being relaxed for a moment. Then sit up, throw the head forward sufficiently to relax the tendons, and rock the head as before; it will now be found that the tendons remain relaxed, showing that tightness of the

*Gray's Anatomy. 2d Amer. edit., p. 256. Phil. 1865.

†Traité d'Anatomie descriptive. Troisième edit., tome deuxième, p. 173. Paris. 1851.

tendons did not conceal action of the muscles in the first experiment, and demonstrating that the *sterno-cleido-mastoid* muscles do *not* "serve to depress the head upon the neck." On bringing the head forward it will be found that it is assisted by the muscles only until the head comes to its centre of balance, when the tendons relax, and remain so even when the chin touches the breast. But if the head is obstructed in this downward movement, these muscles will then assist to bring it down in front and to hold it there. The sterno-cleido-mastoid muscles do not, however, in this rock the head upon the atlas, but bring the atlas forward. Neither are they "extensors of the head" in the sense indicated by the books, which seems at first sight to accord more with their insertions back of the centre of the condyles. But the insertion is so peculiar that it requires careful consideration to determine how the muscles affect the head when the sternal and clavicular portions of both sides are in action. The mastoid process is always below the superior curved line upon which the back part of the muscle is inserted. When the process is large it may be more than half an inch below it, although much less when the process is small, as in childhood before the cells are developed. Moreover, the uniformity of position between the mastoid processes and the condyles horizontally is not met with in their vertical relation, the condyles being on some skulls more than half an inch lower than the mastoid processes, while on others the processes are as much below the condyles, the large proportion being between these extremes. These variations go far to show that the sterno-mastoid muscles are not intended to rock the head backward; for when the mastoid process is much lower than the condyles, and especially when it is large and projects forward somewhat, to correspond to the direction of the muscle, it follows that as the head is pulled down by the *trapezii*, etc., the mastoid processes go upward and forward; consequently, if the *sterno-cleido-mastoids* were to act to bring the head down behind, the portion on the mastoid process—the strongest part of the muscle—would hold the head down in front, probably as much as that on the occipital bone would pull it down behind. But their action can be tested by lying down so as to remove the necessity for action of the muscles to hold the atlas. In this position (care being taken not to lift the atlas or neck) the *sternal* portion of the muscles will not act in concert with the other muscles to rock the head back, even if the whole weight of the body is thrown upon the back of the head, and I have been unable to find any action in the clavicular portion, although the action of this part of the muscle is so delicate and prompt that it can be distinctly felt when the foot is raised in walking, the head and body being then thrown over to the other side to restore the balance. Further, when the *sterno-cleido-mastoid* and the *splenius* of the same side are acting in concert to pull the head down to the shoulder, no backward movement of the head is discoverable. *This* is conclusive, for both these muscles having similar insertions, if one rocks the head back the other must, and their combined action would be manifest if they exerted it.

It has been shown that this muscle acts as a lateral *flexor*, in connection with the *splenius* of the same side, but only when the head is obstructed, and then generally by its clavicular portion, the sternal acting only in extreme necessity. It is now seen that it does not flex the head down in front, that is upon the atlas at all, and that its action as an extensor of the head cannot be demonstrated. The proper function of the *sterno-cleido-mastoids* when acting in concert is to give anterior support to the top of the spine, the *splenii* muscles giving posterior support. This may be easily proved by sitting down and watching the tendons. When the head is back of its centre of support both the sternal and clavicular tendons are tightened, when rising they become tenser until the head is started, as it comes into balance they relax. On sitting down the tendons tighten to check the head as it goes back out of balance. Sudden forward movements tighten them until the head is in motion, they then slacken as the head is forward of the centre and the atlas supported by the *splenii* muscles. If the head is in balance any pressure upon the forehead brings the muscles into action to preserve it. The action of the muscles in these movements is but a modification of the service rendered by them in raising the head from the horizontal position, in doing which the muscles at first support more than the weight of the head; for in supporting the mastoid processes they support the atlas, and make it a fulcrum between the bulk of the head and the counter-balance at the other end of the lever; but as the body comes upright and the head into balance, the strain upon the sterno-mastoid muscles gradually diminishes, until the head is held by the posterior muscles, when the spine bears all the weight.

[A reference to the figure will render this explanation more apparent. The same figure also illustrates the action of the muscles of the lower jaw, and confirms the opinions expressed in the subsequent portions of this paper.]

The hyoid bone, in addition to the muscles which pass to it from parts above the lower border of the jaw, gives attachment to others which pass up the front of the neck below the jaw. Of these the *sterno-thyroid* arises close to the centre of the posterior surface of the upper bone of the sternum, and falling back somewhat as it passes up, is inserted into the side of the thyroid cartilage, from whence the *thyro-hyoid* (appearing like a continuation of the preceding) goes up and is inserted into the body and greater cornu of the hyoid bone. The *sterno-hyoid* arises from the sternum and end of the clavicle, and is inserted into the lower border of the body of the hyoid bone. It is separated considerably from its fellow at its origin, but crosses the sterno-thyroid and approaches it in the middle of its course; it leaves the front of the thyroid cartilage uncovered.

The *omo-hyoid* arises from the upper border of the scapula, and occasionally from the transverse ligament which crosses the supra-scapular notch. It passes across and up the side of the neck, to be inserted into the body of the hyoid bone. It crosses over the *scaleni* and *thyro-hyoid*, but under the *trapezius* and *sterno-cleido-mastoid* muscles. It is a doubled-bellied muscle, united by a tendon which is held down by a process of the deep cervical fascia. The first portion is nearly horizontal in its course; but underneath the sterno-mastoid muscle, where the cervical fascia passes around the tendon, it turns up so that the second portion is nearly vertical in its course to the hyoid bone. These are the directions of the muscle when at rest, but when active it approaches the line of its attachments, and the cervical fascia is drawn upward and backward.

The *digastric*, another double-bellied muscle, has peculiar relations with the preceding. It arises from the digastric notch, on the inner side of the mastoid process of the temporal bone, and passes downward, forward, and inward, to the side of the hyoid bone, where its rounded tendon (after passing through the stylo-hyoid muscle) is held by an aponeurotic loop in connection with the side of the body of the hyoid bone above the insertion of the omo-hyoid. The muscle then passes forward, and is inserted into a large depression on the inner side of the lower border of the jaw close to the symphysis. The tendon which divides the posterior and longer belly from the anterior gives off a large aponeurotic layer, which is attached to the body and great cornu of the hyoid bone; it is termed the *supra-hyoid aponeurosis.* It forms a strong layer of fascia between the anterior portions of the two muscles, and a firm investment for the other muscles of this region. This muscle is peculiar in not being inserted into the hyoid bone, but attached to it by a loop; this allows the muscle to act without interfering too much with the hyoid bone. The *digastric* muscle has not, however, that freedom which is attributed to it as a reflected cord, for its aponeurotic connection with the hyoid bone and adjoining muscles prevents it from sliding through the loop which attaches it to the hyoid bone.

The digastric muscle is set down as drawing the hyoid bone backward and forward in deglutition, and as depressing the jaw by acting as "a reflected cord." These services are inconsistent with each other and with the anatomy of the parts. If it were fixed so as to draw the bone backward and forward, it could not slide and be of service as a "reflected cord" sufficiently to lower the jaw. To do the latter the anterior belly should be inserted higher up the jaw, while a long unrestricted tendon of the muscle should run through a fixed loop on the lower border of the hyoid bone, which last should also be freed from the styloid ligaments, and be drawn down half way to the sternum every time the jaw opened wide, and proportionally for less opening.

In respect to the united action of both bellies drawing the head backward, it is only necessary to say that the origin of the digastric is partly in front of a line drawn across just behind the condyles of the occipital bone; it could not, therefore, draw the head back appreciably even if its insertion were directly under its origin. It is consequently a mistake to suppose it can do so when its direction forward is so nearly horizontal. In fact this muscle is the great agent in drawing the head *forward*. The posterior belly slants down to the hyoid bone, but the anterior is nearly horizontal in its course, and when the muscle acts it tends to the line of its attachments by drawing or endeavoring to draw the hyoid bone upward, unless the jaw is much depressed, when, as the muscle is straight, or nearly so, it has no power to raise the hyoid bone. But in several important services the digastric acts in concert with the omo-hyoid. In this way the muscles passing from the hyoid bone to the front of the jaw, including the anterior belly of the digastric, are as effectually antagonized as if a powerful muscle passed from each side of the hyoid bone to the opposite cervical vertebræ, with the advantage of greater length of muscle to contract, and easier adaptation to the movements of the jaw; and the muscles in front of the hyoid bone act, when necessary, in alternation with the omo-hyoid and the posterior belly of the digastric. Frequently, however, the anterior belly of the digastric acts with the posterior belly and the omo-hyoid, for they keep the head upright. In doing this the omo-hyoid muscle and the posterior belly of the digastric draw or hold the hyoid bone back, while the anterior belly of the digastric brings in the chin, and the *temporal* and other elevators of the jaw draw the head forward; in this way the digastric acts on a long lever, as the head rocks on a centre, but a little below the entrance of the external ear. These muscles are always active during forward or backward movements of the body or head. They do for the head what the sterno-mastoid muscles do for the spine, and their action can be felt easily with the finger, in sitting down or rising up, etc. They are also powerful rotators of the head, and the action of the omo-hyoid is singularly quick in sudden turns of the head (as with the sterno-mastoid muscles), the digastric being useful in assisting to keep the hyoid bone up in place, it being held laterally by the aponeurosis and probably by the mylo-hyoid muscle.

If the end of the finger is placed just behind the origin of the *cleido-mastoid* during these movements, the omo-hyoid will be felt rising above the clavicle, and carrying the cervical fascia upward and backward; and if a finger is placed behind the mastoid process so as to cover the end of the digastric notch, the digastric muscle will be felt acting in concert with the omo-hyoid, and the anterior belly can be felt between the jaw and hyoid bone. The peculiar attachment of the digastric can now be appreciated, as the hyoid bone is left sufficiently free in its various movements, although it is at the same time the centre of control and support to the head. The importance of this support to the head can hardly be overestimated, for the weight of the head beyond the atlas must be balanced This the digastric and omo-hyoid muscles do effectually by acting upon the jaw, which is a lever in length below the atlas, of about one-third of the height of the head above it. The points from which these muscles act are the mastoid process and the shoulder; the vertex of their angle being in the hyoid bone, from which they draw in the chin; in this direction they are very active and powerful. They not only balance the head in locomotion and leave the other muscles free to act in deglutition, vocalization, and articulation, but give them important assistance.

The following quotations show the opinions entertained as to the action of the muscles which move the lower jaw:

*Gray's Anatomy* says: "The temporal, masseter and internal pterygoid raise the lower jaw against the upper with great force. The two latter muscles, from the obliquity in the direction of their fibres, assist the external pterygoid in drawing the lower jaw forward upon the upper, the jaw being drawn back again by the deep fibres of the masseter, and posterior fibres of the temporal. The external pterygoid muscles are the direct agents in the trituration of the food, drawing the lower jaw directly forward, so as to make the lower jaw project beyond the upper. If the muscle of one side acts, the corresponding side of the jaw is drawn forward, and the other condyle remaining fixed, the symphysis deviates to the opposite side. The alternation of these movements on the two sides produces trituration."*

*Todd & Bowman's Physiological Anatomy*, part iii. p. 539, says: "The *external pterygoid* neither raises nor depresses the lower jaw."

My own views as to these muscles differ materially on some points from those expressed in these quotations. The position of the *masseter* is too well known to need particular description. The fibres of the deep portion have a more perpendicular direction than those of the superficial portion, the last passing backward as much as downward. The deep portion draws the jaw upward and backward, the superficial portion upward and forward. The *internal pterygoid* has the same general direction as the superficial portion of the *masseter*, excepting that as it rises from the pterygoid fossa it passes considerably outward to reach its insertion on the inner side of the ramus and angle of the jaw. It has, therefore, not only the upward and forward motion of the superficial portion of the masseter, but also lateral power over the jaw.

These muscles not only raise the jaw and teeth, when cutting with the incisors and crushing with the molars, but are also the main movers of the jaw in trituration. In cutting they bring the jaw forward bodily, in triturating they exert more forward and lateral action on one side than on the other; by this the jaw is thrown over to the opposite side and then *drawn in and carried out continuously on that side*, and not carried over to the other side. It is a mistake to suppose that trituration of the food is effected by the alternate action of the muscles of both sides. When the jaw and teeth are perfect, the teeth of one side only are used, until the muscles tire, perhaps, and then

* Gray's Anatomy, Descriptive and Surgical. 2d American Edition. p. 252. Phila., 1865.

those of the other side are resorted to. If the teeth are tender, badly placed, or deficient on one side, trituration is performed on the other only.

The *temporal* muscle which covers so large a portion of the side of the head, and is strongly inserted into the inner surface, apex and anterior border of the coronoid process of the jaw, pulls its insertion upward and backward, and assists the *masseter* and *internal pterygoid* muscles in cutting, crushing and triturating the food.

The *external pterygoid* is a short, thick muscle, which arises from the pterygoid ridge on the great wing of the sphenoid, and the portion of bone included between it and the base of the pterygoid process, from the outer surface of the external pterygoid plate and the tuberosity of the palate and superior maxillary bones. It arises in two portions separated by a short interval; they both pass outward and backward and are inserted into a depression in front of the neck of the condyles of the lower jaw, and into the corresponding part of the interarticular fibro-cartilage. The separate portions join and form one muscle previous to their insertion, the middle fibres being horizontal; but as the origin of the muscle is very wide vertically, the upper portion descends in passing back, while the lower ascends. This strong and beautiful muscle has, from its peculiar situation, great influence over the jaw. The origin being more internal than the insertion, gives the muscle control over the condyles laterally, by which they are held firmly in the glenoid cavities, and the great strength of the muscle tends to keep the condyles from being driven back by falls, or blows upon the chin, which otherwise might occur easily, as the glenoid fossæ are very shallow.

A more prominent service of this muscle is when it brings the condyle forward either on its own side alone, as in trituration, or with its fellow of the opposite side, as in cutting by the incisors. In these movements it acts in concert with other motors of the jaw. To consider it especially the triturating muscle is no more correct than to suppose that when triturating with a pestle in a mortar the thumb and forefinger (which are further from the grinding surfaces) are the triturators rather than the fingers below.

The *external pterygoid* muscle controls the upper end of the jaw in concert with the *temporal*, while the muscles attached to the body have especial control below, and by the concerted action of all, power and steadiness are secured in the complicated movements of the jaw, and of the lower teeth against the upper. The *external pterygoid* muscles hold the condyles fixed in any part of the glenoid cavities to which they may be drawn in forward movements of the jaw. For this service they are admirably fitted by the great width of their origin, which enables them to brace the condyles so firmly against the parts above and in front of them, that the jaw is fixed, even when wide open, as firm as if the condyles were hinged in that position.

The *eminentia articularis*, the rounded projection which forms the front of the glenoid fossa, is indispensable to this fixation of the condyle, and as the condyle in coming forward mounts this eminence, the jaw, while going down the front, is also carried down bodily, by which the back teeth of the lower and upper jaws are widely separated. These advantages are gained while the centre upon which the jaw turns is two-fifths down toward the angle. This may be tested by placing the hand and wrist around the back of the neck, applying the end of the finger to the back of the ramus and finding the point upon which it turns, which is probably near the insertion of the internal lateral ligament. By this arrangement the jaw is opened promptly and widely, without interfering with the parts behind, which would be injuriously pressed upon if the condyle remained still and the angle went back far enough to open the jaw wide.

The *external pterygoid* is now to be spoken of in a service in which the action of the muscle is greater in range, frequency and importance than in any other, and in which it has never before been recognized, *that of opening the mouth.* This muscle antagonizes the temporal, masseter and internal pterygoid muscles, and is, especially by its lower head, the *depressor of the lower jaw.* It does this alone when necessary, without assistance from any other muscle. This may be proved by bracing the arm against the breast and applying the thumb firmly to the chin; or better, by placing the jaw flat on the mantel or any fixed support, then on opening the mouth the head will go backward, being drawn back by the external pterygoids whose insertions in the condyles are fixed by support of the jaw.

The action of the external pterygoid muscles in opening the jaw is somewhat similar to their action when the jaw is brought forward to cut with the incisors, the difference in effect being produced by other muscles; the temporals relax in these movements only so far as to permit the jaw to move properly. It may thus be held that the temporals probably act similarly in both movements. The masseters and internal pterygoids, however, must relax in the opening of the jaw instead of assisting to carry it forward as in cutting, while the digastrics, which relax in the forward movement of the jaw, undoubtedly assist to draw the chin back in gaping, vomiting, and in very wide opening of the mouth when voluntary. When not otherwise employed, it is probable that they always assist in opening the jaw, as they are admirably fitted for carrying the chin back when the condyle is going forward. It is, however, clear that they could not carry it back readily if the condyles were not at the same time pulled forward. For when the chin points down very much (as in some persons even with the jaw closed) the digastric muscle forms but a slight angle at the loop, and decided action of it in such cases would tend to keep the condyles of the jaw back in the glenoid cavity from the start, or soon after, and the opening of the jaw would straighten the digastric so much that it would have little or no power to carry the chin back. Even in cases where the anterior belly of the digastric ascends from its loop to the chin, so that it has some vertical direction to favor it, and supposing it to be assisted by strong back fibres in the mylo-hyoid muscle, it is not possible that the condyle could release itself from the pit behind the *eminentia articularis*, and unless it did so and came forward, the jaw could open only to a trifling extent, for it would act as if hinged in the glenoid fossæ. But with the external pterygoids drawing the condyles forward, the jaw opens, as upon a centre in the ramus, and the question arises as to what the jaw really turns upon.

The masseter and internal pterygoid muscles hold the angles very much as if a sling were passed around them, and if it were not for the angle going back it might be said that the jaw turned down upon this centre, supported by these muscles in connection with the *stylo-maxillary ligament*, which is also inserted into the angle of the jaw. Under all the circumstances, however, the insertion of the *internal lateral ligament* around the inferior margin of the dental foramen marks the centre upon which the jaw opens with sufficient exactness.

This will be more clearly understood by recollecting that with the teeth closed, the stylo-maxillary ligament *slants* down to the angle of the jaw; therefore, while the *temporal* muscle relaxes to allow the *external pterygoid* muscle to draw the condyle forward under the *eminentia articularis*, the angle is, at the same time, carried back by the ligament into the perpendicular of its attachment on the styloid process. This allows the jaw to go down bodily, as well as open in front, by which the back teeth of the upper and lower jaws are widely separated, and the size of the oral cavity much enlarged.

The shape of the jaw, whether congenital, or modified by age or accident, may also vary the location of the centre somewhat; but at all ages the condyles must come forward to open the jaw effectually. This can be done only by the *external pterygoid* muscles, whose special office is to move and also fix the condyles, and in connection with the temporal and other elevators, the jaw also. In this way only can the digastrics and associate muscles act efficiently in their most important functions, otherwise they would be disturbed by undue movement of the jaw. In certain cases, however, these muscles, as before stated, assist in moving and holding the jaw.

When the *external pterygoids* throw the jaw wide open, the chin is much below the hyoid bone, but controlled by the anterior bellies of the digastric muscles, the hyoid bone being fixed by the posterior bellies and by the *omo-hyoid* muscles, which can now be felt in action. Consequently the three extremities of the jaw, the chin, the angle, and the condyle, have each diverging or converging muscular support. The *digastrics* go to the chin, the *internal pterygoids* go to the angles, and the *external pterygoids* to the condyles. This arrangement of the muscles in connection with its ligaments holds the jaw firm in all its complicated movements.

It has been shown that the *sterno-cleido-mastoid* muscles give anterior support to the atlas, while the splenii and other muscles give posterior support, and that in combination they support it laterally. This support is so complete that the head is held securely on the atlas in all positions.

These muscles, however, do not *rock* the head upon the atlas, but in all positions the lower jaw is the lever which controls it in its backward or forward movements. When the head is in line with the neck and body, it is controlled by power acting upon the jaw, at right angles to the neck. When the head is thrown back so as to be at an angle to the neck, the muscular power is applied to the jaw more in a line with the neck or in certain modifications of it. Thus the powerful muscles which are attached to the occipital bone, behind the condyles, and act upon the head vertically, are antagonized by muscles which act upon the jaw, principally in a horizontal direction, and the head is effectually controlled, notwithstanding the little power of the muscles attached to the occipital bone in front of the condyles.

The lower jaw is the great lever by which the head is held upright, that is forward, and also flexed upon the neck.

These explanations of the physiological action of the muscles which control and influence the lower jaw, prepare the way for the diagnosis of its fractures.

(*To be continued in the June number of this Journal.*)

## Figures and Tables

**Figure f1:**